# How Norwegian casualty clinics handle contacts related to mental illness: A prospective observational study

**DOI:** 10.1186/1752-4458-6-3

**Published:** 2012-04-20

**Authors:** Ingrid H Johansen, Tone Morken, Steinar Hunskaar

**Affiliations:** 1 National Centre for Emergency Primary Health Care, Uni Health, Uni Research, Kalfarveien 31, 5018, Bergen, Norway; 2 Department of Public Health and Primary Health Care, University of Bergen, Box 7800, 5020, Bergen, Norway

**Keywords:** After-hours care, Mental health services, Emergency medical services, Primary healthcare, Coercion

## Abstract

**Background:**

Low-threshold and out-of-hours services play an important role in the emergency care for people with mental illness. In Norway casualty clinic doctors are responsible for a substantial share of acute referrals to psychiatric wards. This study’s aim was to identify patients contacting the casualty clinic for mental illness related problems and study interventions and diagnoses.

**Methods:**

At four Norwegian casualty clinics information on treatment, diagnoses and referral were retrieved from the medical records of patients judged by doctors to present problems related to mental illness including substance misuse. Also, routine information and relation to mental illness were gathered for all consecutive contacts to the casualty clinics.

**Results:**

In the initial contacts to the casualty clinics (n = 28527) a relation to mental illness was reported in 2.5% of contacts, whereas the corresponding proportion in the doctor registered consultations, home-visits and emergency call-outs (n = 9487) was 9.3%. Compared to other contacts, mental illness contacts were relatively more urgent and more frequent during night time. Common interventions were advice from a nurse, laboratory testing, prescriptions and minor surgical treatment. A third of patients in contact with doctors were referred to in-patient treatment, mostly non-psychiatric wards. Many patients were not given diagnoses signalling mental problems. When police was involved, they often presented the patient for examination.

**Conclusions:**

Most mental illness related contacts are managed in Norwegian casualty clinics without referral to in-patient care. The patients benefit from a wide range of interventions, of which psychiatric admission is only one.

## Background

Low-threshold and out-of-hours services like casualty clinics, emergency rooms and emergency departments play an important role in the emergency care for people with mental illness [1-3]. In Norway casualty clinic doctors are responsible for 38-63% of acute referrals to psychiatric wards [4-6]. As in many other countries [7-10], overcrowding of emergency wards is a problem. In Norway the casualty clinics’ high share of acute referrals has nourished a popularly held belief that inadequate service provision at casualty clinics contribute to the overload of emergency specialist in-patient services and a high use of coercion [5,11,12]. The need of reducing casualty clinic referrals is a recurrent theme in governmental policy documents [11-13], and there is an ongoing debate regarding alternative organisation of emergency psychiatric care [11,12,14-16].

Currently, Norway has a strict two-tiered healthcare system. General practitioners (GPs) serve as gatekeepers for all secondary care, including psychiatric specialist care. No patients can present themselves directly to a hospital. A patient in need of voluntary or involuntary psychiatric care always has to be assessed by a GP for hospital referral. When in need of emergency care during office-hours, patients contact their regular GP’s surgery directly. Out-of-hours (4.00 pm – 8.00 am, weekends and public holidays) patients have to contact casualty clinics organised by the municipality and staffed by GPs. In the initial contact with the casualty clinics, nurses assess the patients’ needs and initiate the appropriate response. Possible responses are advice from a nurse or different types of contact with a GP, i.e. telephone advice, consultation, home-visit or emergency call-out. In situations where an initial assessment by the GP will delay immediate access to specialist life-supporting treatment vital to the patient’s survival, the patient will be transported directly to a somatic hospital. However, there are no direct transports to psychiatric hospitals. Although casualty clinics are intended to be an emergency service, the majority of contacts are of non-urgent nature [17].

Given the popularly held belief of inadequate service provision at Norwegian casualty clinics, the results of a recent study [6] comparing emergency psychiatric admissions from casualty clinic doctors, regular GPs, doctors in medical hospitals, and doctors from other parts of the secondary services in psychiatry, are rather surprising. There were no significant difference in the proportion of emergency admissions that could have been handled in alternative ways and only small differences were found in the characteristics of patients referred from the different agents. Nevertheless, the same study showed that casualty clinic referrals had significantly more use of police assistance and involuntary care than referrals from other agents [6]. Thus a further focus on the casualty clinics is warranted. The findings also suggest that casualty clinics see patients in a rather serious general condition, and that aggressive behaviour is a problem. They thus resonate with international studies reporting worries over own security as a major issue in out-of-hours primary care [18-23], a worry which often has been associated with the care for mentally disturbed patients [19,21,24,25].

Most studies with relevance for out-of-hours care in Norway have studied a patient population already filtered through primary care [4-6]. Little is known about patients retained in primary care. The presentation rate of mental illness at Norwegian casualty clinics seems to be low [26-28], but these estimates of prevalence are somewhat uncertain as diagnoses from the chapter P of the diagnostic system International Classification for Primary Care 2^nd^ edition (ICPC-2) [29] are used as a proxy for mental illness. This approach has unknown validity. Hence we have limited knowledge of the actual prevalence of Norwegian casualty clinic contacts related to mental illness, the filtering process on the way to specialist psychiatric care, and the treatment these patients are given in out-of-hours primary care. The present study used a cohort design to prospectively identify casualty clinic patients with problems related to mental illness including substance misuse. We investigated which care these patients received at the casualty clinics, to which extent they were referred onwards to specialist services and which diagnoses they were given. We also investigated involvement of the police.

## Methods

We used a predefined cohort of seven casualty clinics known as the Watchtowers, whose monthly activity reports are used to monitor emergency primary healthcare activities in Norway [30]. The participating casualty clinics are purposely selected to give a representative sample of Norwegian casualty clinics. The Watchtowers manually record information about all successive contacts to the clinic, including age and gender of the patient, time of contact, priority degree given and first action taken [30]. In this study we added information on the further course of patients’ contact with the casualty clinic. Assessment, initiated treatment, diagnoses and onward referrals are documented by the doctors on call in electronic medical records (EMR). A tailor-made computer program retrieved this information anonymously. The Watchtowers differed in which computer software they used for EMR, and the five users of the dominating software were invited to participate in the study. All the invited clinics consented, but one clinic was later excluded due to lack of local IT-support. The excluded casualty clinics were all rural and had a total population of approximately 38000 inhabitants.

The four participating casualty clinics covered a population of approximately 180000 inhabitants. They ranged from a small rural casualty clinic where the GP on call was supported by a nurse during daytime and evenings, to larger city based casualty clinics with other health personnel and up to three GPs on duty around-the-clock.

Earlier studies have shown seasonal variation in casualty clinic contacts related to substance misuse or mental illness, with July as the most aberrant month [26,31]. This study was therefore performed in winter and spring to enhance the representativeness of the sample. In the period from January throughout May 2010 the regular Watchtower recordings were expanded to include whether the attending nurse considered the contact to be related to mental illness or substance misuse problems. These recordings are henceforth referred to as the *Watchtower log*.

As Norwegian regulations limits the possibility to trace individual patients through the healthcare system, we had to separately identify patients with contacts related to mental illness or substance misuse problems seen by GPs to obtain information on the GPs contact with the patients. Thus a pop-up window was activated whenever the GP closed a patient’s EMR and if a consultation, home visit or emergency call-out had been recorded for that patient the same day. The window contained a question asking whether today’s contact with the patient was related to mental illness or substance misuse. To remove the window and be able to continue to work with other medical records, the GP had to tick off one of the following statements: ‘no’, ‘yes, substance misuse’, ‘yes, mental illness’ or ‘yes, both mental illness and substance misuse’. During the study period the casualty clinics had information posters about the study on display in their waiting room. The posters included information about the patients’ right to refuse participation. In cases where the patient did not want to participate in the study, the GP would tick off that ‘the patient does not want to participate in the study’. All GP generated responses were recorded in an anonymous log which included information of the patient’s age and gender. This log will henceforth be referred to as the *GP generated log*.

Unfortunately, the tailor-made computer-program producing the pop-up window stopped working in periods with maintenance of the computer servers and in periods with high activity on the system. Several of the casualty clinics shared servers with local hospitals or regular GPs’ surgeries, thus this interference typically happened during daytime. Altogether this resulted in a substantial loss of GP-registered contacts, especially in the largest casualty clinic where an upgrade of the computer system resulted in the loss of all cases over several weeks. All types of contacts were equally affected independent of their relation to mental illness or substance misuse, and thus no selection bias was introduced.

For contacts judged to be related to mental illness or substance misuse an additional log stored identification of the contacts. At the end of the study period a specifically designed computer program retrieved the EMR text related to these contacts. No information that could directly identify the patient was ever retrieved, and all text was completely anonymous. Based on information available in the text, the following was recorded for each contact: age and gender of the patient, interventions at the casualty clinic beyond standard consultation, onwards referrals, diagnoses given, involvement by the police and reports of dangerous situations. All the casualty clinics used ICPC-2 for diagnoses [29]. Results from this part of the study will be referred to as the *extracts from the electronic medical records*.

The study was approved by the Regional Committee for Medical Research Ethics and the Norwegian Social Science Data Services. The Ministry of Health and Care Services gave permission to use patient information in the study.

All three data sets were analysed descriptively using SPSS 15.0. Means are quoted as mean ± standard deviation. Group differences were tested with Pearson’s chi-squared test or Student’s *t* test.

## Results

### The Watchtower log

During the study period 28527 contacts were recorded at the casualty clinics. The attending nurse judged 715 contacts (2.5%) to be related to mental illness or substance misuse. In contacts related to mental illness or substance misuse 52.6% (n = 361) of the patients were men, compared to 46.2% (n = 12804) for other contacts (p = 0.001). The mental illness or substance misuse patients had a mean age of 38.5 ± 15.0 years, whilst the other patients had a mean age of 33.6 ± 26.2 years (p < 0.001).

Table 1 shows initial contacts to the casualty clinics by period of day, priority grade given, type of primary action taken, and by whether the contact reason was related to substance misuse or mental illness. Contacts related to substance misuse or mental illness differed from other contacts by being more urgent and more frequent during night time. The contacts were frequently handled by nurses only and they resulted less frequently in a consultation with a GP.

**Table 1:** Initial contacts by substance misuse/mental illness relatedness and by day distribution, urgency and action taken

	**Substance misuse/mental illness**		**Others**		
	**n**	**%**	**n**	**%**	**p-value**
**Time of day, n = 28527**					<0.001
08.00-15.29	162	22.7	10381	37.3	
15.30-22.59	313	43.8	14121	50.8	
23.00-07.59	240	33.6	3310	11.9	
**Priority grade, n = 28387**					<0.001
Acute	31	4.6	802	2.9	
Urgent	249	36.7	8523	30.8	
Not urgent	398	58.7	18384	66.3	
**Action taken, n = 28417**					<0.001
Contact with nurse					
Telephone advice	209	30.8	4949	17.8	
Consultation	13	1.9	473	1.7	
Contact with GP					
Telephone advice	78	11.5	2514	9.1	
Consultation	314	46.2	17481	63.0	
Home visit	9	1.3	235	0.8	
Emergency call-out	19	2.8	499	1.8	
Other	37	5.4	1587	5.7	

Initial patient contacts to triaging nurses in the four casualty clinics (n = 28527) by judged relation to substance misuse or mental illness, and by period of day, assessed priority grade, and type of primary action taken. Group differences were tested with Pearson’s chi-squared test.

### The GP generated log

**Figure 1:**
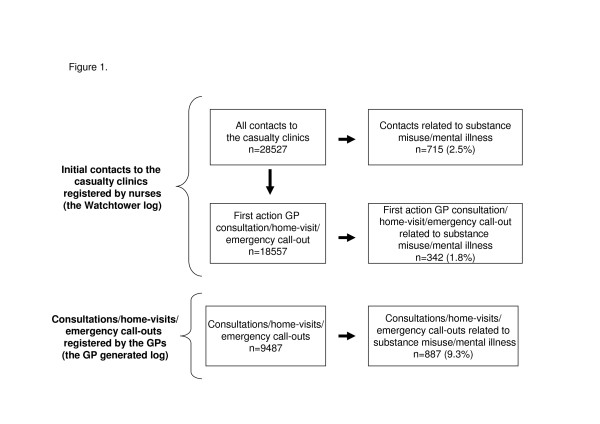
**Total number of registered contacts and share with identified relation to substance misuse/mental illness.** Share of contacts with identified relation to substance misuse/mental illness in nurse registered initial contacts to the casualty clinics (n = 28527) and GP registered consultations, home-visits and emergency call-outs (n = 9487). GP registered contacts where the patient refused to participate in the study are excluded (n = 266). First action GP consultation/home-visit/emergency call-out are initial contacts where the nurses decided that the patient had to be seen by a GP.

During the study period 9753 cases were registered in the GP generated log. The GP generated log thus included 52.6% of the initial contacts to the casualty clinic where the nurses had chosen a consultation, home-visit or emergency call-out as first action. In 266 cases (2.7%) the patient refused to participate in the study, thus 9487 cases were available for further analysis. The non-participant group consisted of 48.5% men (n = 129) and the patients had a mean age of 44.7 ± 24.1 years. The non-participating patients did not differ significantly from the participating patients regarding gender (p = 0.628) or age (p = 0.240). For an overview of contacts registered by nurses and GPs available for further analysis, see Figure 1.

The GPs judged 887 of the eligible contacts (9.3%) to be related to substance misuse or mental illness. The gender distribution differed significantly between the subcategories (p < 0.001), with high proportions of men in subcategories related to substance misuse (Table 2). The patients with contacts related to only substance misuse were younger than the other patients (Table 2). All subgroups related to mental illness or substance misuse had relatively more night time contacts than other types of contacts (p < 0.001) (Table 2).

**Table 2:** GP registered contacts by substance misuse/mental illness relatedness, and by gender, age and day period

	**Total**		**Men**		**Age**		**Time of day**					
							**08-15.59**		**16-22.59**		**23-07.59**	
**Contact related to**	**n**	**%**	**n**	**%**	**mean**	**SD**	**n**	**%**	**n**	**%**	**n**	**%**
Substance misuse	305	3.2	209	68.5	34.6	15.6	56	18.4	95	31.1	154	50.5
Mental illness	375	3.9	147	39.2	40.0	18.3	79	21.1	202	53.8	94	25.1
Substance misuse/mental illness combined	207	2.2	123	59.4	39.1	13.6	58	28.0	81	39.1	68	32.9
Other issues	8600	90.7	3979	46.3	43.5	22.4	2727	31.7	4680	54.4	1193	13.9
**All**	**9487**	**100.0**	**4458**	**47.0**	**43.0**	**22.0**	**2920**	**30.8**	**5058**	**53.3**	**1509**	**15.9**

The GP registered patient contacts in the study period (n = 9487) by whether the contact was related to substance misuse or mental illness, and by share of total, gender and age distribution, and distribution over the day. SD denotes standard deviation.

### The extracts from the electronic medical records

Of the 887 contacts where information was retrieved from the EMRs, 20 cases (2.3%) were excluded due to no or uncertain information about the type of contact. Another 14 cases (1.6%) could only be analysed for diagnoses as information was missing on the interventions at the casualty clinic.

The most common interventions at the clinic differed between the subcategories (Table 3). Frequent interventions were laboratory tests, consulting others regarding the case, the administering or prescription of medication, and minor surgical treatment. In general, there were small differences in the interventions described for cases concluded at the casualty clinic compared to cases referred onwards (data not shown).

**Table 3:** The most common interventions in GP registered contacts related to substance misuse or mental illness

	**Total** **n = 853**		**Substance abuse** **n = 292**		**Mental illness** **n = 361**		**Mental illness/** **substance abuse** **n = 200**		
**Intervention**	**n**	**%**	**n**	**%**	**n**	**%**	**n**	**%**	**p-value**
Laboratory tests	198	23.2	86	29.5	78	21.6	34	17.0	<0.01
Consulted others regarding treatment	143	16.7	32	11.0	70	19.3	41	20.5	<0.01
Given medication	128	15.0	35	12.0	63	17.5	30	15.0	0.15
Prescriptions	113	13.2	23	7.9	64	17.7	26	13.0	0.001
Minor surgical treatment	77	9.0	51	17.5	15	4.2	11	5.5	<0.001
Observation in casualty clinic	55	6.4	22	7.5	17	4.7	16	8.0	0.20
Sick leave	19	2.2	5	1.7	11	3.0	3	1.5	0.38

The most common interventions in GP-contacts related to substance misuse or mental illness at the casualty clinics (n = 853). Only interventions beyond dialogue between the patient and the GP are included. Percentage denotes percentage of all patients in given category. Group differences were tested with Pearson’s chi-squared test.

Half of the patients received all necessary treatment at the casualty clinic (Table 4). Treatment at a specialist level was needed more frequently for patients presenting problems related to substance misuse and mental illness combined compared with patients presenting problems related to only substance misuse or mental illness. A third of patients seen by GPs were referred to in-patient treatment, and many were admitted to other than psychiatric wards. Only one patient was referred to addiction treatment. Of 131 patients admitted to psychiatric services, 50 (38.2%) were admitted involuntarily.

**Table 4:** Use of onwards referral in GP registered contacts related to substance misuse or mental illness

	**Total** **n = 853**		**Substance abuse** **n = 292**		**Mental illness** **n = 361**		**Mental illness/** **substance abuse** **n = 200**		
	**n**	**%**	**n**	**%**	**n**	**%**	**n**	**%**	**p-value**
**No onwards referral**	**428**	**50.2**	**154**	**52.7**	**190**	**52.6**	**84**	**42.0**	**0.03**
**Onwards referral**									
**Out-patient treatment**	**121**	**14.0**	**45**	**15.4**	**54**	**15.0**	**22**	**11.0**	**0.33**
Somatic services	49	5.7	38	13.0	9	2.5	2	1.0	<0.001
Regular GP	34	4.0	6	2.1	20	5.5	8	4.0	0.08
Psychiatric services	31	3.6	1	0.3	23	6.4	7	3.5	<0.001
Addiction treatment	1	0.1	0	0.0	0	0.0	1	0.5	N/A
Others	6	0.7	0	0.0	2	0.6	4	2.0	N/A
**In-patient treatment**	**290**	**34.0**	**87**	**29.8**	**117**	**32.4**	**86**	**43.0**	**<0.01**
Psychiatric wards	131	15.4	6	2.1	78	21.6	47	23.5	<0.001
Medical wards	114	13.4	53	18.2	30	8.3	31	15.5	0.001
Surgical wards	31	3.6	22	7.5	5	1.4	4	2.0	<0.001
Community based wards	7	0.8	1	0.3	3	0.8	3	1.5	N/A
Other somatic wards	6	0.7	4	1.4	1	0.3	1	0.5	N/A
Addiction treatment	1	0.1	1	0.3	0	0.0	0	0.0	N/A
**Police custody**	**19**	**2.2**	**6**	**2.1**	**4**	**1.1**	**9**	**4.5**	**0.03**

Onwards referral from the casualty clinics in GP registered contacts related to substance misuse or mental illness (n = 853). The columns add to more than 100% because some patients were referred to several services. Percentage denotes percentage of all patients in given category. Group differences were tested with Pearson’s chi-squared test. N/A marks when Pearson’s chi-squared test was non-applicable due to low expected cell-count.

The police was involved in 148 contacts. In 123 of these (83.1%) the police presented a patient for examination. In the remaining 25 contacts (16.9%) the police was alerted by the casualty clinic staff. The police assisted in 34 admissions to in-hospital treatment, whereof four were to medical and surgical wards, and the remaining 30 were involuntary admissions to psychiatric wards. In 29 of the 30 involuntary psychiatric admissions the patient was presented for examination by the police. Threatening behaviour by patient or relatives was mentioned in 32 of the GP registered contacts related to mental illness or substance misuse. This included one episode where health personnel were physically abused. Police was involved in 17 of the contacts with display of threatening behaviour. In 12 contacts the patient had arrived with the police, and in five contacts the police were alerted by the casualty clinic staff. In eight of the contacts involving threatening behaviour the patient was involuntarily admitted to a psychiatric hospital.

The most commonly used diagnoses varied between the 3 subcategories (Table 5). In addition to reflecting substance abuse, the diagnoses in the substance-misuse-only group were often related to acute injuries like cuts and concussion. Far from all cases had been given diagnoses from the chapter P of ICPC-2. One or more diagnoses from chapter P had been given in 32.1% of cases related to substance misuse only (n = 96), 58.6% of cases related to mental illness only (n = 214) and 70.4% of cases related to both mental illness and substance misuse (n = 143).

**Table 5:** The most frequently used diagnoses in GP contacts related to substance misuse or mental illness

**Substance misuse (n = 299)**			**Mental illness (n = 365)**			**Mental illness/substance misuse (n = 203)**		
**ICPC-code**		**%**	**ICPC-code**		**%**	**ICPC-code**		**%**
**P16**	Acute alcohol abuse	14.4	**P76**	Depressive disorder	8.8	**A84**	Poisoning by medical agent	11.8
**S18**	Laceration or cut	12.0	**P74**	Anxiety disorder/anxiety state	7.7	**P19**	Drug abuse	9.4
**P19**	Drug abuse	10.0	**P99**	Other psychological disorders	7.1	**P99**	Other psychological disorders	8.4
**N79**	Concussion	5.7	**P27**	Fear of mental disorder	5.5	**P15**	Chronic alcohol abuse	7.9
**A99**	Unspecified general disease	5.4	**P77**	Suicide or suicide attempt	5.5	**P76**	Depressive disorder	7.9
**D01**	Abdominal pain	4.3	**A84**	Poisoning by medical agent	4.7	**P16**	Acute alcohol abuse	6.4
**N80**	Head injury other	4.0	**P02**	Acute stress reaction	4.4	**P74**	Anxiety disorder/anxiety state	6.4
**P15**	Chronic alcohol abuse	3.7	**P73**	Affective psychosis	4.4	**P98**	Psychosis unspecified	5.9
**A11**	Chest pain	2.3	**P98**	Psychosis unspecified	4.4	**P77**	Suicide or suicide attempt	4.9
**L76**	Sprain/strain of joint	2.3	**P72**	Schizophrenia	3.8	**P02**	Acute stress reaction	4.4
**L18**	Musculoskeletal injury	2.3	**A11**	Chest pain	3.6	**P29**	Unspecified psychological symptom	3.4
	All other diagnoses	61.2		All other diagnoses	66.0		All other diagnoses	57.6

The 11 most commonly used ICPC-2 diagnoses at the casualty clinics for patients presenting problems judged by GPs to be related to mental illness or substance misuse. The columns add to more than 100% because patients were given up to four diagnoses.

### Comparison of the casualty clinics

Table 6 shows main characteristics of the individual casualty clinics and sums up key results for each individual casualty clinic. There were marked differences between the casualty clinics in judged urgency level of the initial contacts and number of GP contacts related to mental illness. Other parameters were rather similar, for example share of GP contacts related to substance misuse and share of GP contacts resulting in a referral to in-patient treatment.

**Table 6:** Descriptive background information and key results by casualty clinic (A-D)

	**A**		**B**		**C**		**D**				
**Descriptive information**											
Number of municipalities participating in the casualty clinic	1		10		1		1				
Number of inhabitants 01.01.10	18680		88997		8360		67305				
Description of community	Mostly rural		Urban and rural		Rural		Urban				
									**Total**			
**Key results**	**n**	**%**	**n**	**%**	**n**	**%**	**n**	**%**	**n**	**%**	**p-value**	
**Nurse registered initial contacts**												
Total number of contacts	5493		10818		2456		9760		28527			
Contacts related to substance misuse or mental illness	139	2.5	198	1.8	32	1.3	346	3.5	715	2.5	<0.001	
Judged acute or urgent	43	32.3	101	53.7	12	38.7	124	38.0	280	41.3	<0.001	
Handled by nurse	38	28.6	42	22.5	5	16.1	137	41.8	222	32.7	<0.001	
Home-visits/emergency call-outs	7	5.3	8	4.3	5	16.1	8	2.4	28	4.1	<0.01	
**GP registered contacts**												
Total number of contacts	1486		3123		806		4072		9487			
Contacts related to substance misuse	40	2.7	123	3.9	23	2.9	119	2.9	305	3.2	0.05	
Contacts related to mental illness	73	4.9	138	4.4	15	1.9	149	3.7	375	4.0	0.001	
Contacts related to substance use and mental illness combined	49	3.3	67	2.1	12	1.5	79	1.9	207	2.2	<0.01	
**Extracts from medical records**												
Total number of extracts	158		314		44		337		853			
Total referrals to in-patient treatment	46	29.1	108	34.4	14	31.8	122	36.2	290	34.0	0.47	
Admissions to psychiatric ward	16	10.1	52	16.6	3	6.8	60	17.8	131	15.4	0.05	
Involuntary admissions	2	1.3	19	6.1	2	4.5	30	8.9	53	6.2	0.01	

Percent denotes percentage of relevant contacts. Group differences were tested with Pearson’s chi-squared test.

## Discussion

In this study the GPs judged 9.3% of their patient contacts to be related to mental illness or substance misuse, and most contacts were handled without referral to in-patient care. A wide range of interventions took place at the casualty clinics and a substantial share of patients received counselling by nurse only. When involved, the police often presented a patient for examination. Less than 70% of relevant patients were given diagnoses reflecting mental illness or substance misuse.

Nearly 40% of initial contacts to the casualty clinics were handled by nurses, and three quarters of GP face-to-face contacts were handled by GPs without in-patient referrals. Thus more than 80% of all casualty clinic contacts were handled within the casualty clinic system by use of ambulatory care. The low rate of referrals to inpatient care was consistent across individual casualty clinics, and occurred despite the higher judged urgency of mental illness or substance misuse contacts compared to other types of contacts. The wide range of interventions reported and specialist referrals used suggest that meeting a generalist in the initial contact with the healthcare system could benefit the patients. Even in the patient group with contacts related to only psychiatry a significant number of patients received or were referred to non-psychiatric treatment. Studies of GPs’ participation in out-of-hours emergency healthcare have observed a switch from hospitalisation to ambulatory care [32-36], and there are indications that when presented with the same emergency cases, specialists tend to admit more patients than generalists do [37-39]. The use of GPs as gatekeepers might optimise the use of available specialist resources both in terms of the general filtering to specialist care and filtering into specific sub-groups of specialist care. However, other study designs are needed to judge the appropriateness of current treatment and referrals at casualty clinics.

Earlier findings of three times more police assistance in emergency psychiatric referrals from casualty clinics compared to referrals from other agents have raised issues about how casualty clinics handle these patients [6]. In this study police involvement was mentioned in 148 out of 853 contacts (17.4%), and when involved, the police frequently presented the patient for examination. These findings probably suggest that the situations are rather accentuated before the patients arrive at the casualty clinic. Higher relative rates of more serious diagnoses at casualty clinics compared to at regular GP’s surgeries [31], combined with high estimated urgency and use of emergency call-outs, further strengthen this impression. If the casualty clinics’ involvement in emergency psychiatric admissions really is a problem, then the most efficient initiatives to reduce their involvement might be to prevent crises from escalating by improving early crisis detection and prevention [40]. Consequently, alternative care provision when the crisis has already escalated, for example crisis resolution teams, will have limited effect on emergency admissions [41,42], although they undoubtedly contribute to better healthcare services for patients not necessarily in need of an emergency admission.

In this study we wanted to mirror the clinical experience of casualty clinic staff, thus the selection of the cohort relied on subjective assessment by nurses and GPs. There was a marked discrepancy of the GP- and nurse-reported shares of contacts related to mental illness. This might partly be due to an awareness effect as the GPs were actively asked about this relation for each contact by a pop-up window, whilst the nurses ticked off a box among others in a standard registration form. The discrepancy might also reflect the limited information available to nurses in the initial assessment of patients compared to the information GPs possess after a face-to-face patient encounter. Less than 70% of patient contacts marked by GPs as related to mental illness or substance misuse were given diagnoses from the chapter P of ICPC-2. Limited validity of routinely set diagnostic codes has previously been reported [43,44], and our findings suggest that the prevalence of mental illness related contacts out-of-hours are underestimated in former Norwegian reports. Our study also identified a substance misuse related group of patients mainly presenting with injuries or intoxications. These patients were rarely given diagnoses from the chapter P of ICPC-2 and would therefore have been missed in earlier studies [26,27,31]. Our findings thus further highlight the need for contextual sensitivity and interpretative caution when studying prevalence by use of routinely set diagnostic codes.

## Conclusions

This study shows that substantial triage takes place at the casualty clinics, and that four out of five patients contacting the casualty clinic for mental illness or substance misuse related problems are helped by the casualty clinic staff without further referral to in-patient treatment. Many of the patients needed other than psychiatric specialist care. It is therefore likely that patients in their initial emergency contact benefit from the generalists’ broad range of qualifications, and that thepresence of GP-based casualty clinics may reduce the overload of emergency psychiatric wards, rather than induce it.

## Abbreviations

GP: General practitioner; EMR: Electronic medical record.

## Competing interests

The authors declare that they have no competing interests.

## Authors’ contributions

IHJ conceived of the study. She collected, analysed and interpreted the data, and she drafted and revised the manuscript. TM helped interpret the data and revised the manuscript critically for important intellectual content. SH contributed to the design of the study and revised the manuscript critically for important intellectual content. All authors read and approved the final manuscript.
